# Oxidative Stress in Polycystic Ovarian Syndrome and the Effect of Antioxidant N-Acetylcysteine on Ovulation and Pregnancy Rate

**DOI:** 10.7759/cureus.17887

**Published:** 2021-09-11

**Authors:** Jasmine K Sandhu, Ahsan Waqar, Ashish Jain, Christine Joseph, Kosha Srivastava, Olive Ochuba, Tasnim Alkayyali, Sheila W Ruo, Sujan Poudel

**Affiliations:** 1 Obstetrics and Gynecology, California Institute of Behavioral Neurosciences & Psychology, Fairfield, USA; 2 Family Medicine, California Institute of Behavioral Neurosciences & Psychology, Fairfield, USA; 3 Internal Medicine, California Institute of Behavioral Neurosciences & Psychology, Fairfield, USA; 4 Urology/Obstetrics and Gynecology, California Institute of Behavioral Neurosciences & Psychology, Fairfield, USA; 5 Neurology, California Institute of Behavioral Neurosciences & Psychology, Fairfield, USA; 6 Internal Medicine, Marmara University, Istanbul, TUR; 7 Pathology, California Institute of Behavioral Neurosciences & Psychology, Fairfield, USA; 8 General Surgery, California Institute of Behavioral Neurosciences & Psychology, Fairfield, USA; 9 Psychiatry and Behavioral Sciences, California Institute of Behavioral Neurosciences & Psychology, Fairfield, USA

**Keywords:** n-acetylcysteine, pcos, oxidative stress, antioxidants, infertility

## Abstract

Polycystic ovarian syndrome (PCOS) is an endocrinological condition that leads to infertility in many females. N-acetylcysteine (NAC), a novel antioxidant, is being used as an adjuvant to treat infertility in females suffering from PCOS. This review aims to evaluate oxidative stress in females suffering from PCOS and assess whether the anti-oxidizing properties of NAC are beneficial in enhancing the rate of ovulation and pregnancy in infertile PCOS females. A literature search was conducted manually on PubMed and Google Scholar databases using the following keywords: “N-Acetylcysteine,” “PCOS,” “Oxidative stress,” “Antioxidants,” and “infertility” alone and/or in combination for data collection. The studies were manually screened and, after applying inclusion-exclusion criteria, 32 studies consisting of 2466 females of the reproductive age group are included in this review. Our review revealed that females suffering from PCOS tend to show elevated levels of inflammatory markers and a decrease in antioxidant capacity. When used in combination with clomiphene citrate or letrozole, NAC increases ovulation and pregnancy rate in infertile females suffering from PCOS and positively affects the quality of oocytes and number of follicles ≥18mm. Moreover, its side effect profile is low. It also results in a mild increase in endometrial thickness in some females. Future studies on a large sample size using NAC alone are highly recommended to evaluate its role as a single-drug therapy for treating infertility in females suffering from PCOS.

## Introduction and background

Polycystic ovarian syndrome (PCOS) is a multi-system endocrinological disorder affecting females of the reproductive age group characterized by clinical and biochemical abnormalities such as menstrual irregularities, hyperandrogenism, infertility, hyperinsulinemia, and multiple ovarian cysts. In recent years, the incidence of PCOS is rapidly growing due to changes in lifestyle, diet, and stress. Its prevalence is variable, ranging from 2.2-2.6%, and has affected 116 million females globally, according to WHO estimates in 2012 [1]. A survey conducted in India in 2020 showed that 16% and 11% of females in the age groups 20-29 years and 30-44 years, respectively, suffer from PCOS [2].

**Figure 1:**
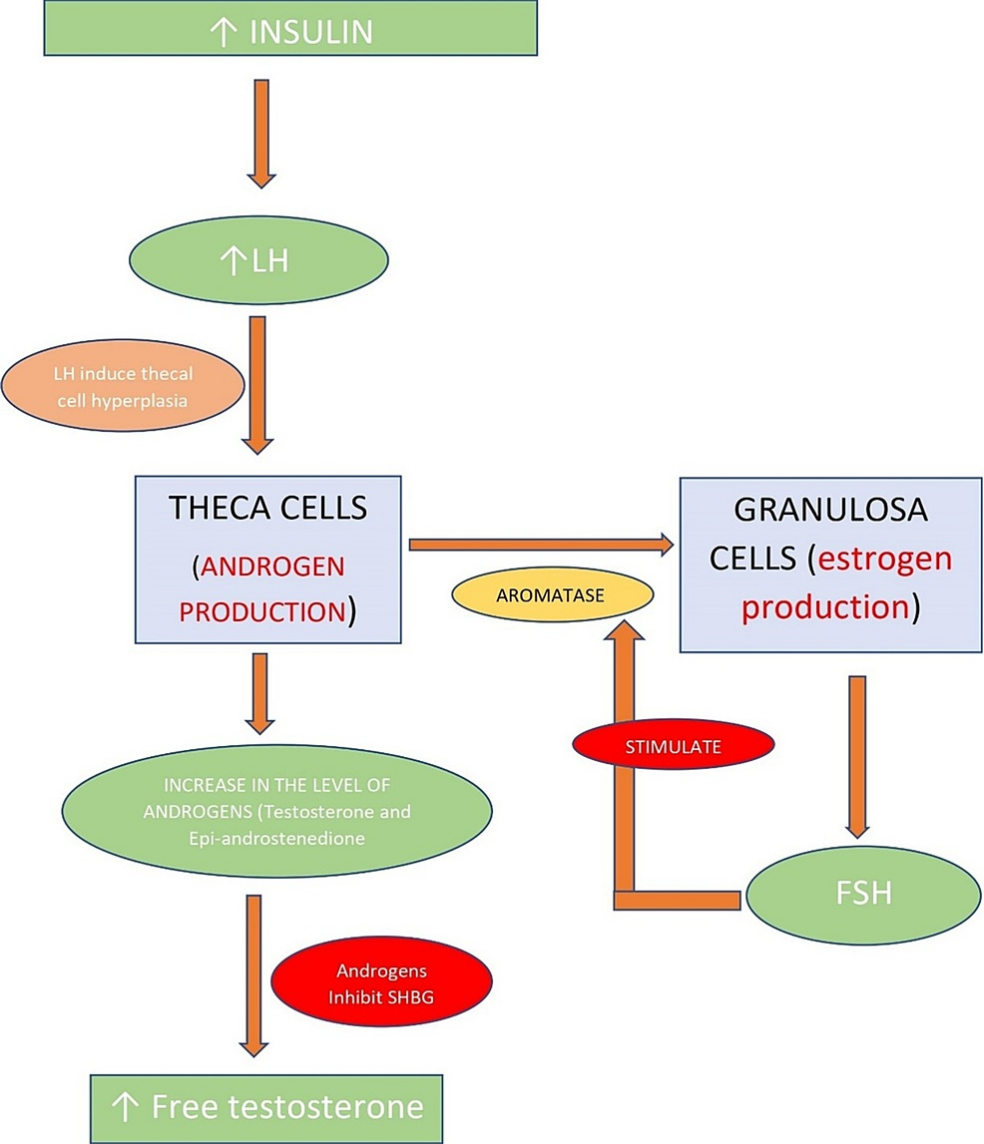
Sequence of events in the pathogenesis of PCOS LH: luteinizing hormone; FSH: follicle-stimulating hormone; SHBG: sex hormone-binding globulin

PCOS is diagnosed using the Rotterdam Criteria-2003, which includes two of the following features: oligo/amenorrhea, anovulation, infertility; hyperandrogenism (clinical or biochemical signs and symptoms); and polycystic ovaries (12 or more) [3]. It may be set in early adolescent life but clinically manifests in childbearing age with long-term complications such as type 2 diabetes mellitus, hypertension, hyperlipidemia, endometrial cancer, and cardiovascular disorders [4]. Early identification and treatment can help mitigate some of these adverse metabolic effects. PCOS is a multifactorial disorder caused by both genetic and environmental factors. It is mainly seen in obese females with resultant insulin resistance [5]. Hyperinsulinemia elevates luteinizing hormone (LH), which then induces thecal cell hyperplasia resulting in androgen overproduction. Androgens inhibit hepatic production of sex hormone-binding globulin (SHBG), leading to increased free testosterone levels in the body, as illustrated in Figure 1, which present clinically as excessive facial hair and acne [4]. Androgens also inhibit the growth of the dominant follicle and prevent apoptosis of smaller follicles leading to cyst formation in the ovaries. The hormonal imbalances seen in females with PCOS are decreased follicle-stimulating hormone (FSH) and FSH/LH ratio, increase in the level of LH, fasting insulin, estrogen, free testosterone, and a mild increase in prolactin [4].

Lifestyle changes such as weight loss and smoking cessation are effective in treating PCOS. Additionally, oral contraceptives, FSH, LH, and gonadotropin-releasing hormone (GnRH) analogs, metformin, letrozole, and laparoscopic ovarian drilling (LOD) are also used to treat PCOS. Metformin, an insulin-sensitizing agent, is extremely helpful in obese females with insulin resistance [4]. Infertility is a significant problem faced by females suffering from PCOS. It is defined as an inability to conceive after a year of regular and unprotected intercourse [6]. About 20% of infertility is attributed to anovulation caused by PCOS [4]. Various treatment modalities are employed to cure infertility, but the success rate remains low. Clomiphene citrate (CC) is the first line of treatment to induce ovulation, but it is futile in many patients, along with ovarian hyperstimulation syndrome (OHS) seen in 10% of cases [4]. Recently, antioxidants such as N-acetylcysteine (NAC), Myo-inositol, and micronutrients have been used in addition to the above medications to improve the chances of getting pregnant [4].

NAC, the N-acetyl derivative of natural amino acid L-cysteine, is a known antioxidant [7]. It has been commonly used for acetaminophen toxicity and as a mucolytic for decades [7]. It has also been used to treat cystic fibrosis, atherosclerosis and its complications, and malignancy [8]. There are several theories behind the mechanism of action of NAC. First, it interacts with electrophilic groups and scavenges free radicals such as hydrogen peroxide and superoxide with the help of its thiol side chain [7,9]. Second, it acts as a source of cysteine (Cys) for glutathione (GSH) synthesis and, third, it breaks disulfide linkages, hence acting as a mucolytic [7,9]. The elevated glutathione levels in the cells result in insulin binding to carrier proteins, which helps glucose uptake and utilization [8]. This leads to a decrease in insulin levels and thus alleviates the leading cause of PCOS. The antioxidant properties of NAC have been utilized in multiple clinical trials conducted on infertile PCOS females to improve oocyte quality and enhance pregnancy and ovulation rate [8].

This literature review aims to provide information on the effectiveness of NAC on oocyte quality, follicular size, pregnancy, and ovulation rate in infertile females suffering from PCOS. The PubMed and Google Scholar databases were searched manually using the following keywords: “N-Acetylcysteine,” “PCOS,” “Oxidative stress,” “Antioxidants,” and “Infertility” alone and/or in combination to find studies relevant to the topic of research. A total of 1957 studies were obtained, which were later screened based on title and abstract. We included systematic reviews, traditional reviews, randomized control trials (RCT), clinical trials, case-control studies, cross-sectional studies, and books. We included only those studies from Google Scholar published in the last three years (2018-2021). This review article excluded animal studies, grey literature, case series, case reports, and studies on the male population. Thirty-two studies consisting of 2466 females are included in this review after applying inclusion-exclusion criteria, including information from a book and websites. The study consists of two systematic reviews, 12 traditional reviews, seven RCTs, two controlled clinical trials, seven case-control studies, and two cross-sectional studies.

## Review

Oxidative stress and PCOS

Oxidative stress is defined as an imbalance in the level of oxidants and antioxidants in the body and is measured with the help of the following parameters: total antioxidant status (TAS), total oxidant status (TOS), and oxidative stress index (OSI) [10]. OSI is the percentage ratio of TOS to TAS [10]. Oxidant-antioxidant imbalance is seen in females suffering from PCOS, and this oxidative stress is due to various metabolic abnormalities such as obesity, hyperinsulinemia, and dyslipidemia [11]. Obesity is seen in 40-50% of females with PCOS, which results in lipid catabolism and free radical production, ultimately leading to oxidative stress [12]. Hyperglycemia results in an increased reactive oxygen species (ROS) generated from mononuclear cells [13]. ROS are oxygen-free radicals produced by reducing molecular oxygen and generated as byproducts of aerobic respiration and metabolism [13]. ROS plays a crucial role in various bodily functions by regulating cell growth, differentiation, progression, and cell death and, thus, their balance is required in the body. ROS results in activation of tumor necrosis factor-alpha (TNF-α) and an increase in the level of nuclear factor-kappa B (NF-κB), an inflammatory transcription factor [13]. Inflammatory markers stimulate androgen production in ovaries leading to hyperandrogenism and, thus, links PCOS with inflammation [14]. TNF-α is a known mediator of insulin resistance [15]. Therefore, a vicious cycle is created in the ovarian epithelium, resulting in the formation of cysts, low-quality ovarian follicles, and infertility [13]. Antioxidant enzymes such as superoxide dismutase (SOD), catalase, and glutathione peroxidase protect cells against toxic ROS. Studies conducted on females with PCOS showed alterations in the levels of inflammatory markers, some of the above-mentioned protective enzymes, and antioxidant status compared to females with no ovarian pathology.

 A case-control study conducted by Enechukwu et al. on 50 PCOS patients revealed a significant decrease in the total antioxidant capacity (TAC) (p<o.oo1) and activity of SOD (p<0.001) when compared with 50 females with normal ovarian function [16]. Moreover, there was a significant elevation in the level of malondialdehyde (MDA) [16]. MDA, a known marker of oxidative stress, is the final product of poly-unsaturated fatty acid peroxidation [17]. Another case-control study by Bannigida et al. on 100 PCOS patients also revealed a significant elevation in MDA levels (p<0.001) compared to 100 females in the control group [18]. A case-control study by Shahrokhi and Naeni compared the level of TAC and MDA in 60 females with PCOS to 90 healthy females [12]. The results showed no statistically significant difference in the mean TAC between the two groups; however, MDA levels were significantly higher in PCOS patients [12]. A case-control study by Sak et al. on 94 females (46 PCOS females and 48 healthy controls) revealed that TOS and OSI are significantly higher in the PCOS group compared to control (p=0.002 and p=0.004 respectively) [10]. It also showed a significant decrease in TAS in the PCOS group [10]. A study by Liu et al. also showed significantly higher levels of TOC (p<0.001) and OSI in the PCOS group compared to the control [19]. A case-control study by Sulaiman et al. showed a higher level of glutathione peroxidase, and glutathione reductase in 51 females with PCOS (p>0.05) compared to 45 healthy females [20]. It also showed lower level of glutathione (p=0.006) and TAC (p>0.05) in PCOS group [20]. A study by Jeelani et al. also showed a significant increase in the level of MDA and SOD and a decrease in TAC in 95 females suffering from PCOS compared to the control group [21]. A cross-sectional study conducted by Artimani et al. on 21 females with PCOS undergoing Intracytoplasmic sperm injection (ICSI) showed a higher concentration of follicular fluid levels of inflammatory markers including interleukin-6 (IL-6), IL-8, and TNF-α and lower concentrations of IL-10, an anti-inflammatory interleukin compared to the control group [14]. Moreover, the level of TAC and Thiol groups were lower in the PCOS group [14]. Another cross-sectional study by Moti et al. on 30 females suffering from PCOS also revealed decreased total antioxidants in PCOS patients compared to 30 females with normal ovarian function [22].

The findings of the studies show that chronic inflammation and oxidative stress are one of the causative factors of PCOS. The elevated levels of oxidative markers further aggravate the pro-inflammatory status in PCOS females [21]. To treat females suffering from PCOS, we need a drug with the capacity to modulate the levels of inflammatory markers and antioxidants. NAC elevates the level of glutathione, a known antioxidant and, thus, increases total antioxidant capacity. In addition, the thiol-free side chain of NAC increases the level of thiol in females treated with NAC. NAC acts as an anti-inflammatory molecule by scavenging free radicals such as hydrogen peroxide and superoxide and inhibiting the release of TNF-α and other interleukins from phagocytic cells by down-regulating the activity of NF-κB [8]. Hence, it lowers inflammation and oxidative stress in the ovarian epithelium.

Effect of NAC on oocyte quality 

Meiotic and cytoplasmic maturation of oocytes is negatively impacted by inflammation and oxidative stress, which results in decreased fertilization and implantation [23]. A negative correlation is seen with TOC and high-quality embryo and blastocyst formation in the PCOS group in a case-control study by Lei et al. (p<0.05) [19]. Another study by Lei et al. on infertile females undergoing in vitro fertilization and embryo transfer (IVF-ET) showed that females suffering from PCOS have lower fertilization, clinical pregnancy, implantation rate, and a higher rate of miscarriage compared to females with tubal factor infertility (p>0.05) [24]. Moreover, ROS in granulosa cells is significantly higher (p<0.05) in the PCOS group [24]. The increased ROS in PCOS females leads to increased apoptosis of granulosa cells, which affects the oocyte quality and, hence, reduces the effectiveness of IVF-ET pregnancy results in females suffering from PCOS [24]. Oocytes require various growth factors for proper growth and maturation of follicles during reproductive cycles. The primary oocyte-secreted factors (OSFs) are growth differentiation factor-9 (GDF-9) and bone morphogenic factor-15 (BMF-15) [25]. These factors can be used to evaluate the quality of oocytes [25]. C-kit is another OSF and studies show a relationship between c-kit with mitogen-activated protein kinases (MAPK) pathway and phosphoinositide 3-kinase (P13K) pathway essential in the development of follicles [26]. A double-blind placebo-controlled clinical trial was conducted in the IVF unit in Iran on 80 females aged 25-35 years diagnosed with PCOS based on the Rotterdam criteria planning to undergo ICSI. The subjects were divided into four groups, with 20 patients in each group. Group one received a placebo; Group two received metformin (MET); Group three received NAC, and Group four received MET + NAC. Results revealed a significant elevation in the expression of GDF-9 and a decrease in expression of c-kit in unfertilized mature oocytes in the patients receiving NAC compared to other groups. The study concluded that NAC interferes with the MAPK pathway and thus decreases c-kit levels [25]. Additionally, the antioxidant nature of NAC has an inhibitory effect on NF-κB and, thus, increases the expression of GDF-9. A similar study by Cheraghi et al. on 60 PCOS women in Iran showed a significant decrease in the number of abnormal and immature oocytes and an increase in healthy oocytes (p<0.05) in the NAC recipients [27]. There was also a significant decrease in the level of MDA in NAC patients (p<0.02) [27].

These studies show that NAC has a positive effect on the growth and maturation of oocytes and hence, can improve pregnancy rates by producing good quality oocytes for fertilization. Therefore, we could employ N-acetylcysteine’s impact on these ovarian factors to improve the quality of oocytes in females suffering from PCOS. 

Effect of NAC on ovulation and pregnancy

To evaluate the effect of NAC on ovulation and pregnancy rate, we assessed seven RCTs, out of which four were double-blind studies [28-34]. The studies consisted of a total of 1207 females suffering from PCOS diagnosed based on the Rotterdam criteria. The trials were conducted by randomly dividing people into different treatment groups receiving either clomiphene citrate or letrozole along with NAC or a placebo to compare the effectiveness of NAC (Table 1). 

**Table 1:** Characteristics of studies included: type, period, country, number of patients, treatment, and inclusion criteria. RCT: randomized controlled trial; NAC: N-acetylcysteine; CC: clomiphene citrate; MET: metformin; PCOS: polycystic ovarian syndrome; ORS: oral rehydration solution; LOD: laparoscopic ovarian drilling

STUDY ID	STUDY TYPE	STUDY PERIOD	COUNTRY	NUMBER OF PATIENTS	TREATMENT	INCLUSION CRITERIA
Teimouri et al. [28]	RCT	2018	Iran	317	Group A: letrozole 5mg + NAC 1200mg; Group B: letrozole 5mg	Presence of PCOS, normal thyroid hormones and prolactin levels, infertility duration of at least one year, one patent fallopian tube, normal semen analysis of partner
Hassan et al. [29]	RCT	April 2018 - Jan 2019	Egypt	150	Group A: CC (100mg/day) + NAC (1.2g/day); Group B: CC (100mg/day) + placebo (ORS)	Presence of PCOS, both fallopian tubes patent, normal semen analysis of partner
Mostajeran et al. [30]	Double-blind RCT	2015-2016	Iran	130	Group A: letrozole 5mg + NAC 1200mg; Group B: letrozole 5mg + placebo (ORS)	Presence of PCOS, age 20-35 years, BMI < 35kg/m², patent fallopian tubes, normal semen values of partner
Maged et al. [31]	RCT	September 2012 -March 2014	Egypt	120	Group A: CC only; Group B: CC + NAC; Group C: CC + MET	Presence of PCOS diagnosed on the basis of Rotterdam Criteria
Salehpour et al. [32]	Double-blind placebo-controlled RCT	Jan 2008 – Dec 2009	Iran	180	Group A: CC (100mg/day) + NAC (1.2g/day); Group B: CC (100mg/day) + placebo	Presence of PCOS diagnosed on the basis of Rotterdam Criteria
Nasr A [33]	Double-blind placebo-controlled RCT	Jan 2005 - June 2007	Egypt	60	Group A: NAC (1.2g/day); Group B: placebo	Females with CC-resistant PCOS who underwent unilateral LOD
Rizk et al. [34]	Double-blind placebo-controlled RCT	March 2002 - Nov 2003	Egypt	150	Group A: CC (100mg/day) + NAC (1.2g/day); Group B: CC (100mg/day) + placebo	Females with CC-resistant PCOS undergoing infertility treatment, Age 18-39 years

An RCT by Teimouri et al. was conducted in Iran [28]. Treatment was administered for five days to 317 infertile females suffering from PCOS, following which 5000 units of human chorionic gonadotropin (hCG) were injected intramuscularly if a minimum of one follicle measured ≥18mm on vaginal ultrasound. They were advised to have intercourse, and on the 12th day after the injection, the level of β-hCG was checked to assess for pregnancy. Results showed a statistically significant increase in pregnancy rate in NAC-treated patients (p=0.046). Twenty-three females (14.6%) got pregnant in group A (NAC-treated) compared to 12 females (7.5%) in group B in the trial. There was no statistically significant change in follicular size and endometrial thickness in the intervention group [28]. An RCT was conducted by Hassan et al. on 150 females suffering from PCOS diagnosed based on the Rotterdam criteria [29]. The females were injected with 10,000 units of hCG on the 12th day of the cycle. The results showed a significant increase in ovulation (Group A (NAC treated): 40% v/s Group B: 24%) and pregnancy rate (p=0.007) in females receiving NAC. Twenty-eight females (18.7%) got pregnant in group A compared to 12 females (8%) in group B [29]. Mostajeran et al. conducted a double-blind RCT on 130 females to study the effect of adding NAC to letrozole for treating PCOS [30]. Results revealed a significant increase in ovulation rate (p=0.032) and pregnancy rate (p=0.045). Twelve females got pregnant in group A (NAC treated) compared to four females in group B [30]. Moreover, the size of follicles is also significantly improved in NAC treated group (p=0.007) [30]. Another RCT by Maged et al. also showed similar results concerning follicular size, ovulation rate, and pregnancy rate (p<0.05) in females treated with NAC [31].

Salehpour et al. conducted a placebo-controlled double-blind RCT on 180 females. Results showed a significant increase in follicular size (number of follicles≥18mm) (p<0.001), mean endometrial thickness (p<0.001), ovulation rate (p=0.02), and pregnancy rate (p=0.04), along with zero cases of OHS [32]. Two more placebo-controlled double-blind RCTs were conducted in Egypt on females with CC-resistant PCOS [33,34]. There was a significant increase in ovulation rate (p<0.01) (Group A (NAC treated): 87% v/s Group B: 67%), pregnancy rate (p<0.01) (Group A: 77% v/s Group B: 57%), and live birth rate, and fewer miscarriages in females receiving NAC after LOD in the RCT conducted by Nasr A [33]. The other trial was conducted by Rizk et al. on 150 females with CC-resistant PCOS who also showed improvement in ovulation and pregnancy rate (21.3% got pregnant in group A (NAC treated) compared to zero in group B) along with no cases of OHS with NAC [34]. 

All the clinical trials showed statistically significant improvement in pregnancy (p<0.05), and six trials showed a substantial increase in the rate of ovulation (p<0.05) in females treated with NAC [28-34]. The dominant follicle should be of the appropriate size (≥18mm) for fertilization to occur. Four out of eight studies showed a significant increase in the number of follicles≥18mm in size in the intervention group, and one study had higher appropriately sized follicles in the control group [30-32]. The thickness of the endometrium is another factor determining the success of pregnancy as implantation of blastocyst only takes place in the uterus if the endometrium is of appropriate thickness. Three studies showed a statistically significant increase in endometrial thickness in the females receiving NAC, and one study showed no difference in both groups. Based on these studies, we concluded that NAC serves as an effective treatment modality for infertility issues in females suffering from PCOS. 

A systematic review and meta-analysis of RCTs were also conducted by Thakker et al. in 2014 on 910 females to review the drawbacks and benefits of NAC in women with PCOS [35]. The review concluded that the ovulation and pregnancy rate was three times higher in NAC recipients than in non-NAC recipients, with a higher live birth rate and fewer side effects in the former. However, the studies included were of shorter duration (three months) with a limited number of patients and wide confidence intervals [35].

## Conclusions

Females suffering from PCOS show an increase in the level of inflammatory markers in addition to decreased levels of antioxidants. N-acetylcysteine shows promising results in restoring this oxidative balance and promoting high-quality oocytes with appropriate follicular size. It also increases ovulation and pregnancy rate with fewer side effects such as ovarian hyperstimulation syndrome, commonly seen with other medications. However, most of the trials included in this review were done on a small population group, and NAC was used as an additive to letrozole and clomiphene in treating infertility in PCOS patients. Therefore, there is a need to conduct clinical trials on a large sample size to show better efficacy results. In addition, since the NAC-treated group also has a low side effect profile compared to other groups, tests should be conducted to assess the effect of this drug as single-drug therapy in the future instead of being used as an adjuvant to other medications.
